# Physical Performance Outcomes and Programme Delivery Costs of ELDERPLAY, a Community-Based Multicomponent Exercise and Recreational Intervention for Older Adults: A Non-Randomized Controlled Evaluation

**DOI:** 10.3390/healthcare14203441

**Published:** 2026-10-14

**Authors:** Alberto Salas-Cañizares, Solanger Hernández-Méndez, María Jesús Hernández-López, José Antonio Vera-Pérez, María Ruzafa-Martínez

**Affiliations:** 1 Quality, Research, Training and Teaching Unit, Rafael Méndez University General Hospital, Health Area III, Murcian Health Service (Servicio Murciano de Salud, SMS), Ctra. Nacional 340, km 589, 30817 Lorca, Spainmariaj.hernandez34@carm.es (M.J.H.-L.); 2 Faculty of Health and Social Sciences, University of Murcia, 30100 Murcia, Spain; 3 Faculty of Nursing, University of Murcia, 30100 Murcia, Spain; javerape@um.es (J.A.V.-P.); maruzafa@um.es (M.R.-M.)

**Keywords:** frailty, aged, exercise therapy, recreation therapy, actigraphy, costs and cost analysis, community health nursing

## Abstract

**Highlights:**

**What are the main findings?**

- Participation in the ELDERPLAY programme was associated with improvements in balance, gait performance, lower-limb functional performance and overall physical performance in community-dwelling older adults.
- The programme was also associated with higher daily step counts and was delivered at a mean cost of €92.3 per participant, or €62.3 per participant when the research monitoring devices are excluded.

**What are the implications of the main findings?**

- A brief community programme combining structured multicomponent exercise with recreational activities was delivered in full at a mean cost of €92.3 per participant and offers health professionals a pragmatic option for addressing frailty-related functional decline outside hospital settings.
- Whether the approach can be culturally adapted and scaled within primary care and community services in ageing populations, supporting independence and healthy ageing, remains to be evaluated.

**Abstract:**

**Background/Objectives:** This study examined the association between participation in a community-based multicomponent physical and recreational programme (ELDERPLAY) and changes in physical performance and frailty-related outcomes in adults aged 65 years and older, and estimated programme delivery costs. The study was designed as a quasi-experimental, non-randomized controlled evaluation conducted in a community setting. **Methods:** Community-dwelling older adults (n = 184) were allocated to an intervention group (n = 103) or a control group (n = 81) through voluntary enrolment and geographic matching. Participant enrolment and data collection were carried out between July 2022 and December 2023, after ethics approval had been obtained. The intervention consisted of 20 supervised sessions (two per week) combining Vivifrail-based multicomponent exercises with simple recreational activities. Primary outcomes were physical performance and frailty-related function, assessed using the Short Physical Performance Battery and the Timed Up and Go test. Secondary exploratory outcomes included actigraphy-derived daily step count, heart rate, and sleep parameters, recorded over seven consecutive days at a single intermediate assessment point. Analyses were conducted using analysis of covariance and generalized estimating equations adjusted for cholesterol, COVID-19 vaccination and Barthel Index score. Programme costs were estimated using an activity-based costing approach. **Results:** Compared with the control group, participants in the intervention group showed greater improvement over time in balance, gait performance, chair-stand performance and overall physical performance (time × group interaction: gait performance *p* < 0.001, partial eta squared 0.450; chair-stand performance *p* < 0.001, 0.306; SPPB total score *p* < 0.001, 0.361; Timed Up and Go *p* < 0.001, 0.224). At 10 weeks, the adjusted mean SPPB total score was 9.62 (95% CI 9.25–10.00) in the intervention group and 7.80 (95% CI 7.37–8.23) in the control group, and the adjusted mean Timed Up and Go was 8.85 s (95% CI 8.08–9.62) versus 12.04 s (95% CI 11.15–12.92). Higher daily step counts were also observed in the intervention group; heart rate and sleep parameters were recorded cross-sectionally at a single intermediate assessment point that coincided with the delivery of the programme and are reported only as descriptive values. The programme was delivered at a mean cost of €92.3 per participant, or €62.3 per participant when the research monitoring devices are excluded. **Conclusions:** Participation in a community-based multicomponent exercise programme incorporating recreational activities was associated with improvements in clinically relevant physical performance outcomes in community-dwelling older adults. The programme was delivered in full at a mean delivery cost of €92.3 per participant in a real-world community setting. Because allocation was not randomized, the groups differed at baseline, and outcome assessment was carried out by a single unblinded assessor who designed and coordinated the programme but did not deliver the sessions, these findings are associative and require confirmation in a randomized trial. Trial registration: ClinicalTrials.gov NCT07864831 (retrospectively registered).

## 1. Introduction

The increase in hygiene and sanitary conditions has significantly contributed to the increase in life expectancy in recent decades. The World Health Organization (WHO) estimates that by 2030, one in six people worldwide will be older than 60 years old [1]. The forecasts indicate that ageing is an unstoppable phenomenon. Between 2015 and 2050, it is estimated that the percentage of people aged 60 years or older will almost double, from 12% to 22% [1]. Figures differ according to the age group and the geographical scope considered: the estimates above refer to people aged 60 years or older worldwide, whereas in the European Union the proportion of individuals aged 65 years or older increased from 16.2% in 2003 to 21.3% in 2023 [2]. Spain shows similar figures, and it is estimated that by the year 2072, those older than 65 years of age will comprise 26% of the population [3].

This population ageing, although representing a positive health and social achievement, also presents critical challenges, including the increasing prevalence of frailty. Frailty is considered a multidimensional and dynamic condition, characterized by a loss of functional reserve in diverse physiological systems, which, taken together, results in a lower capacity to cope with stressful situations [4]. Five criteria have been proposed for its diagnosis: a decrease in gait speed, reduction in physical activity, exhaustion, involuntary weight loss, and muscle weakness [5]. These indicators have been validated by diverse studies [6] and allow for early detection that is key to avoiding progression towards dependence.

Frailty affects between 12% and 18% of adults aged 65 years or older in the United States and Europe [7]. In Spain, the figures are very similar, with an estimated 18% of individuals over 65 years of age experiencing frailty [8]. This condition directly affects the quality of life, autonomy, and longevity of older adults, and is associated with an increase in the risk of adverse events: between 1.2 and 2.8 times in falls and fractures, between 1.5 and 2.6 times in physical limitation, between 1.2 and 1.8 times in hospitalizations, and between 1.8 and 2.3 times in mortality [9]. In addition, it implies an increase in the total mean healthcare costs of $3286 and $4653 as compared to the costs of individuals with pre-frailty and robustness, respectively [10].

The model of healthy ageing proposed by the WHO in 2002 promotes a positive and functional approach to ageing [11]. In this conceptual framework, physical exercise is presented as a fundamental tool for preventing and mitigating the effects of frailty [1]. A modality specifically adapted for older individuals is gerontogymnastics, which involves tailoring physical exercise to the functional capacities of this age group. The current recommendations for older adults describe the application of physical activity programmes that include strength, resistance, balance, and flexibility exercises, adapted to the functional level of each individual [12]. Consistent with this, the WHO recommends that adults aged 65 years and over undertake at least 150–300 min of moderate-intensity aerobic physical activity per week, muscle-strengthening activities on two or more days per week, and multicomponent physical activity emphasizing functional balance and strength training on three or more days per week [13]. Experimental studies have shown positive effects on functional capacity, balance, and the reduction in frailty of these types of programmes, which typically lasted an average of 12 weeks and were performed 2 or 3 days a week by older adults with varying levels of frailty [14].

Aside from structured exercise, incorporating traditional games can be an effective strategy for promoting physical activity in older adults. These games, which are orally transmitted from one generation to the next, reflect the culture and values of each community [15]. In the Spanish context they include games such as musical chairs, steal the bacon, petanque, hopscotch, bowling and relay races. They share common elements such as rules, objectives, simple equipment, and defined spaces. Some include roles and narratives that enrich the recreational experience. In addition, they promote physical, cognitive, and social skills [16]. Because they rely on simple rules and readily available materials, traditional games can be implemented with minimal resources, and they have been reported to strengthen respect, cooperation and a sense of community belonging [17]. Likewise, participation in social activities has been shown to be a protective factor against frailty [18].

The effectiveness of diverse types of non-pharmacological, multicomponent interventions on improving factors associated with frailty in older adults is well documented [19]. Scientific research has shown that traditional games significantly contribute to the physical health of individuals, especially children and adolescents [20]. Although the impact on health is promising, there are very few studies that assess the effectiveness of exercise programmes with recreational elements on older adults [21].

Two considerations underlie the incorporation of recreational elements into structured exercise programmes for older adults. First, although multicomponent exercise is effective, adherence and long-term maintenance remain the main obstacles to its sustained delivery in community settings [22]. Second, traditional games are culturally embedded, require minimal equipment and are intrinsically social, which may support participation through enjoyment and group affiliation rather than through prescription alone. The gap addressed by this study is therefore twofold: whether a programme combining standardized multicomponent exercise with adapted traditional games is associated with improvements in physical performance in community-dwelling older adults, and at what delivery cost, given that the available evidence on recreational formats derives largely from children and adolescents.

Therefore, this study aimed to examine the association between participation in the ELDERPLAY physical and recreational programme and changes in physical performance and frailty-related outcomes in people over 65 years of age living in the community. Secondary objectives included evaluating other indicators of physical capacity and sleep, and estimating the delivery costs of this intervention.

## 2. Materials and Methods

### 2.1. Study Design

Quasi-experimental study with a non-randomized control group that evaluated the effects of the physical-recreational programme “ELDERPLAY” over 10 weeks in people over 65 years of age in the municipality of Lorca (Murcia, Spain). This non-randomized study has been reported in accordance with the EQUATOR Network guidance, specifically adhering to the TREND Statement (Transparent Reporting of Evaluations with Nonrandomized Designs); the completed checklist is provided as Supplementary Materials.

Random allocation was not applied. The ELDERPLAY programme was embedded in a municipal gerontogymnastics service promoted by the local council and delivered by the Poncemar Foundation. Enrolment was free, open and voluntary for any resident aged 65 years or over, and participants were already enrolled in these workshops before the study began, as part of the ordinary service provision of the organization. Random reassignment would have required altering the ordinary organization of the programme and restricting access to a health-promotion activity that was already available and had already been accepted by the participants; this was considered neither operationally feasible nor ethically appropriate, since it would have meant withholding an existing community service for research purposes alone. A quasi-experimental design with a non-randomized control group was therefore adopted. Participants were consequently self-selected into the intervention group, and the study should be read as a pragmatic, hypothesis-generating evaluation preceding a randomized trial, with its findings reported in associative rather than causal terms. Accordingly, the TREND statement for non-randomized designs was followed instead.

### 2.2. Study Setting and Sampling

Individuals over the age of 65 years who lived in the community within the municipal boundaries of Lorca, in the Region of Murcia, Spain, were selected. In 2021, this population comprised 14,572 individuals, 6396 men and 8176 women, representing about 15% of the total population, with a life expectancy of 83 years [23]. Preparatory tasks and institutional liaison began in 2022; participant enrolment and data collection were conducted between July 2022 and December 2023, after ethics approval had been obtained from both ethics committees (see Section 2.9), with a 10-week follow-up period per participant.

The selection of participants was conducted through non-probabilistic sampling, by convenience, to ensure representation from all areas of the municipality, including both urban and rural areas. The sampling calculation was performed with the Granmo v.8.0 programme [24]. By estimating a clinically relevant difference of 1 point in the Short Physical Performance Battery (SPPB) and considering a standard deviation of 2.2 points, along with a 10% substitution rate, a total of 80 subjects per group was deemed necessary.

### 2.3. Inclusion and Exclusion Criteria

The inclusion criteria were individuals aged 65 years or older of both sexes, a lack of cognitive deterioration or mild cognitive deterioration, as measured through the Pfeiffer Questionnaire (Pfeiffer score > 7 points), and conserved mobility (absence of incapacitating illnesses that impeded walking with or without support devices, such as a cane). Exclusion criteria included institutionalization in a nursing home and/or regular participation in structured physical exercise outside the study programme (i.e., attending supervised exercise sessions more than twice a week). The use of a walking aid such as a cane was not in itself an exclusion criterion: preserved mobility was defined as the ability to walk with or without a support device, and participants who walked with a cane were eligible. Concurrent physiotherapy was not recorded and was not addressed by the eligibility criteria as applied.

The intervention group was selected from among the users who had voluntarily registered to participate in gerontogymnastics workshops directed by professionals from the Poncemar Foundation, which take place in all areas within the municipality of Lorca. For the selection of the control group, contact was made with the Espartaria Federation of Neighborhood Associations to recruit adults aged 65 years and older living in the same urban neighbourhoods or surrounding districts as the participants in the intervention group. Both groups were recruited concurrently, and follow-up was carried out in parallel in the intervention and control groups.

### 2.4. Study Intervention

The intervention group took part in the physical-recreational training programme named “ELDERPLAY” for 10 weeks, 2 days a week. Each subject received 20 training sessions, with a maximum of 15 participants per session. All intervention sessions were delivered by three qualified sports instructors. Before the start of the programme, the instructors received standardized training on the intervention protocol, the content of each session, the progression criteria and the implementation procedures, all of which had been developed by the principal investigator, in order to ensure homogeneous delivery across groups. The principal investigator designed and coordinated the programme and performed all outcome assessments, but did not deliver the intervention sessions. The duration of each session was 60 min. Each of the sessions was composed of 4 parts: warm-up, physical exercises based on the Vivifrail Project [12], a physical and recreational activity based on adapted versions of traditional games (steal the bacon, musical chairs, relay races, tic-tac-toe, petanque, hopscotch, red light green light, hide and seek, bowling, etc.), and concluding with a stretching exercise.

The multicomponent block followed the Vivifrail framework and was individualized according to the functional level assessed at baseline. Each exercise was performed in two sets of ten repetitions, applied to the strength, balance, mobility and functional tasks included in the session and adapted to the functional capacity of each participant. Rest intervals were one minute between sets of the same exercise and two minutes between the different blocks of the session, in order to facilitate recovery and ensure participant safety.

Exercise intensity was regulated by the professional judgement of the instructor in charge, using the baseline functional assessment (Barthel Index) of each participant as a reference to individualize the workload; neither subjective effort scales, such as the Borg scale, nor heart-rate-based criteria were used. Progression over the ten weeks was gradual and consisted of a progressive increase in the intensity of execution and in training volume, adjusting the number of repetitions and the difficulty of the tasks to the functional evolution of each participant, while the complexity of the games and motor tasks in the recreational component was also increased progressively in order to maintain the physical and cognitive challenge. Progression was supervised by the instructor in charge and adapted to the individual capacities observed during the programme, always within the safety and functionality criteria established by the Vivifrail methodology. The adapted traditional games used in the programme are described in Supplementary Table S2.

Individual attendance at each of the 20 scheduled sessions was not recorded systematically, so neither the mean number of sessions attended and its dispersion nor the proportion of participants reaching a given attendance threshold can be reported, and the effective dose of the intervention actually received cannot be quantified. Compliance at the level of individual exercises and games within each session was likewise not recorded.

Participation was monitored through assessments conducted at the three scheduled time points, and no participant was lost at any of them: all included participants completed the baseline, intermediate, and final assessments. Several organizational features of the programme may have supported participation: sessions were delivered in small groups distributed across the different neighbourhoods and rural districts of the municipality, which reduced travel barriers and facilitated access; the same instructors delivered the programme throughout the ten weeks, favouring a relationship of trust and continuity of follow-up; and participation was voluntary, with no financial incentives or benefits other than the activities included in the programme.

The control group (CG) performed their habitual activities of daily living without any regular physical activity programme.

### 2.5. Variables and Measurement Instruments

A data collection notebook was designed, which included a subset of sociodemographic and clinical variables, primary study variables, and secondary variables.

**Sociodemographic variables:** age, sex, nationality, marital status, family, household composition, and level of education.

**Clinical variables:** Body Mass Index (BMI), chronic diseases, toxic habits (tobacco and alcohol consumption), chronic pain, COVID-19 vaccination, and being diagnosed with COVID-19; cognitive deterioration assessed with the Pfeiffer test, and physical function assessed with the Barthel Index. COVID-19 vaccination was defined as receipt of a COVID-19 vaccine dose during the twelve months preceding the baseline assessment, corresponding to the 2022–2023 booster campaign, and was collected by participant self-report as a dichotomous variable. The figures therefore reflect booster uptake and not primary-series coverage, which exceeded 90% in this age group in Spain during the study period.

**Main variables:** frailty-related physical function is the primary outcome for assessing the effect of the programme, and was measured through the use of the Short Physical Performance Battery (SPPB), which includes three dimensions—balance, gait performance (assessed in this study as the time in seconds taken to walk 4 m), and ability to stand up from a chair—and the Timed Up and Go test (TUG). Each of the three SPPB components is scored from 0 to 4 points, giving a total score ranging from 0 to 12, with higher scores indicating better lower-limb functional performance; scores below 10 points are commonly used to identify limited mobility and frailty risk [8]. Throughout this manuscript, the chair-stand test is reported as a measure of lower-limb functional performance and not as a direct measure of muscle strength, since performance on this test also depends on balance, coordination, movement strategy and confidence.

**Other secondary variables:** sleep quality (Pittsburgh Sleep Quality Index) [25], sleep duration, sleep patterns (deep, light, and REM sleep), heart rate, and number of daily steps, which were monitored through actigraphy. For this, the “Mi Smart Band 6” smartwatch by the brand “Xiaomi” was used (Xiaomi, Beijing, China).

Evidence on wrist-worn consumer devices indicates acceptable validity for step count and heart rate in adults and older people [26,27], whereas sleep-stage classification shows limited agreement with polysomnography, the reference standard [28,29]; no validation of the Mi Smart Band 6 for sleep staging in older adults is available. All sleep-related variables are therefore treated here as exploratory. Participants were instructed to wear the device continuously for seven consecutive days, including night-time periods. Before the recording period, the operation of the device was explained verbally and written instructions for its use were provided, and all participants signed an informed consent form together with a commitment to comply with the monitoring protocol. Given the pragmatic nature of the monitoring, no formal criteria for a valid recording day, based on minimum daily wear time or on the availability of night-time data, were defined a priori; no specific procedure was established for handling non-wear periods; and no imputation of missing data was applied.

Data obtained from the devices were reviewed for physiological plausibility before analysis. Predefined ranges were applied to each variable: 30–220 bpm for heart rate, 100–25,000 for daily step counts, and 180–840 min for total sleep time, with records in which the sum of sleep stages did not match the reported total also flagged. Records falling outside these ranges were treated as measurement or export errors and excluded from the corresponding analyses; no participant was excluded on the basis of a single implausible value in another variable. Heart rate and step data required no exclusions. For total sleep time, five records were excluded: three reported zero minutes, and two exceeded 6000 min, the latter also showing an inconsistency between the reported total and the sum of individual sleep stages. Weekly means were computed from the seven consecutive monitoring days for all remaining records.

All physical performance assessments, including the SPPB and the Timed Up and Go test, were administered by a single assessor, a registered nurse who was the principal investigator of the study and who was responsible for the design and coordination of the programme and for data collection at every time point, but who did not deliver the intervention sessions. The same assessor administered the three assessments for all participants, in order to standardize the measurement procedures and minimize inter-observer variability; the assessor had previous training and experience in the functional assessment of older people and in the administration of the tests used. Because a single assessor performed all the measurements, no inter-rater reliability coefficient was calculated; conversely, outcome assessment was not blinded to group allocation.

### 2.6. Data Collection

At the start of the study, the individuals who met the inclusion criteria were invited to participate, and informed consent was requested. The follow-up period was the same regardless of group (control or intervention). The collection of data took place at three moments in time:

**Time 0 (T0):** At the start of the study, all the measurements were taken of the sociodemographic and clinical variables, Pfeiffer test, and Barthel Index, as well as all the base measurements of the SPPB and TUG frailty tests.

**Time 1 (T1):** The second data recording was performed on the intervention group after ten sessions from the start of the ELDERPLAY training programme, approximately 5 weeks later. In the case of the control group, it was also performed 5 weeks after their incorporation into the study. The SPPB and TUG tests were applied once more, and the quality of sleep was assessed (Pittsburgh); smartwatches were provided to assess sleep patterns, heart rate, and daily steps for seven consecutive days, worn continuously 24 h a day. This was the only actigraphy recording of the study, and it was carried out at the same assessment point, and with the same procedure, in both groups.

**Time 2 (T2):** In the third and final assessment, the SPPB and TUG were applied once again. The evaluation was performed in the intervention group after completing the 20 sessions proposed by the ELDERPLAY programme, that is, 10 weeks after the training started. In the control group, participants were assessed 10 weeks after their inclusion in the study.

### 2.7. Intervention Costs

The costs of the different components of the ELDERPLAY programme were determined using the Activity-Based Costing (ABC) method [30], which is designed to provide a detailed understanding of the costs associated with specific activities and processes. In this study, the costs were classified into equipment (including all exercise activities and a smartwatch for actigraphy), logistics (renting a multi-use room), and personnel costs (salary and transportation). The calculations were based on a structured chronogram in which 103 participants attended a total of 20 sessions. The total costs were calculated by adding the expenses of all sessions conducted during the trial and then dividing by the number of attendees to calculate the cost per person. The analysis was conducted from the perspective of the programme provider and included only the direct costs of delivering the intervention (personnel, transport, facility rental, wearable devices and consumables); costs incurred by participants, informal care and downstream healthcare use were not included. Unit costs correspond to the programme delivery period and are expressed in 2023 euros; given the ten-week horizon per participant, no discounting or price-year adjustment was applied. This is a programme delivery cost analysis: no cost-effectiveness or cost-utility analysis was performed, since neither falls nor health-related quality-of-life utilities were collected as study outcomes.

### 2.8. Data Analysis

Descriptive statistics were used (mean, standard deviation, percentages) to explore the sociodemographic, clinical, and health characteristics of the participants in both groups. To verify the homogeneity between the intervention group and the control group, bivariate analyses were performed using chi-square tests, Student’s *t*-tests, and ANOVAs, depending on the nature of the variables.

The effect of the programme was assessed using two-way ANCOVAs for the quantitative variables (SPPB: gait performance, chair-stand performance, total score; and Timed Up and Go test), considering the time point (T0, T1, T2), and the group (intervention/control). For the dichotomous variables of balance (feet together, semi-tandem, complete tandem), the Generalized Estimating Equations (GEE) model was applied, using a binomial distribution and logit link, considering time (T0, T1, T2), and group (intervention/control). Covariates were selected as baseline variables that showed statistically significant differences between the intervention and control groups in the bivariate homogeneity analyses. The same covariates—cholesterol, COVID-19 vaccination and the Barthel Index—were entered in all models, both in the ANCOVA models for gait performance, chair-stand performance, SPPB total score and Timed Up and Go, and in the GEE models for the balance items. COVID-19 booster uptake was retained as a covariate because it differed markedly between groups at baseline (55.6% in the control group versus 12.6% in the intervention group) and because, in a non-randomized design, such an imbalance may act as a marker of differential health-seeking behaviour and contact with primary care, which may in turn be associated with functional status. The GEE models were fitted specifying an independent working correlation structure together with a robust sandwich (Huber–White) variance estimator for the calculation of standard errors and confidence intervals. This specification was established a priori: as the models included group, time and their interaction on complete and balanced data, the parameter estimates are identical regardless of the correlation structure specified, and the robust estimator ensures the validity of the inference even if that structure were misspecified; the most parsimonious specification was therefore adopted.

Statistical assumptions were checked before modelling for the ANCOVA models. Normality of the distributions and of the model residuals was assessed with the Shapiro–Wilk test, complemented by Q–Q plots, and was not supported; a logarithmic transformation did not restore it. Homogeneity of variances was examined with Levene’s test and Box’s M test, both of which indicated heterogeneity. Homogeneity of regression slopes was examined by testing covariate-by-group interaction terms; heterogeneity was observed only for the Barthel Index in the gait performance model, and stratified sensitivity analyses by Barthel Index category were therefore conducted. Sphericity was examined with Mauchly’s test and was not supported, so within-subjects contrasts were corrected using the Greenhouse–Geisser procedure when epsilon was below 0.75 and the Huynh–Feldt procedure otherwise. Although several of these assumptions were not supported, the ANCOVA results were considered sufficiently robust for the purposes of this evaluation: the F test is comparatively robust to departures from normality and from homogeneity of variance in samples of this size (n = 184) with group sizes of the same order of magnitude, the Greenhouse–Geisser and Huynh–Feldt corrections were applied to the within-subjects contrasts, and the direction and magnitude of the between-group differences were consistent across the four outcomes modelled; the corresponding *p*-values should nevertheless be read as approximate. These assumptions do not apply to the balance items analysed with GEE, which is based on quasi-likelihood and uses a robust variance estimator.

Because allocation was not randomized, the feasibility of a propensity score analysis was formally assessed. Matching would have required discarding most of the sample, and weighting would have produced estimates determined by a small number of observations with extreme weights, owing to the limited overlap of the propensity score distributions; neither procedure was therefore applied. The covariate-adjusted models described above were retained as the analytical strategy. It should be noted that propensity-score methods balance observed covariates only and cannot account for unmeasured determinants of participation, in particular pre-existing motivation towards physical activity, which was not measured in this study.

With respect to the variables obtained through actigraphy (heart rate, sleep time, daily steps), the mean was obtained from the seven days of monitoring. In addition, the mean score for sleep quality was obtained, as assessed through the Pittsburgh questionnaire. The mean scores obtained were compared between the intervention and control groups using Student’s *t*-test for independent samples.

The results were considered significant at *p* < 0.05. All the analyses were performed with IBM SPSS Statistics v26.0 for Mac.

Generative artificial intelligence tools were used only to support language refinement and structural editing of the manuscript. All authors reviewed and approved the final content and take full responsibility for its accuracy, integrity, and originality. No generative artificial intelligence tools were used to create or alter scientific images or figures, or to assist in study design, data collection, analysis, or interpretation.

### 2.9. Ethical Considerations

The study was conducted in accordance with the Declaration of Helsinki. It received ethics approval from the Research Ethics Committee of Health Area III of the Murcian Health Service (CEI-2022-04-20, approved on 5 May 2022) and from the Research Ethics Committee of the University of Murcia (ID 3992/2022, approved on 20 June 2022). Participant enrolment and data collection began after ethics approval had been obtained from both committees; the reference to 2022 as the start of the project reflects preparatory tasks and institutional liaison, whereas the inclusion of participants started in July 2022. Before participating, the subjects were informed both orally and in writing about the objectives and the methodology of the ELDERPLAY programme, and all participants provided written informed consent before participation. The data collected were anonymized to protect the identity of the participants. No economic compensation was offered, and adverse effects were not recorded. At the end of the intervention, the control group was offered information about how to join the physical activity sessions.

## 3. Results

### 3.1. Sociodemographic and Clinical Characteristics

**Figure 1:**
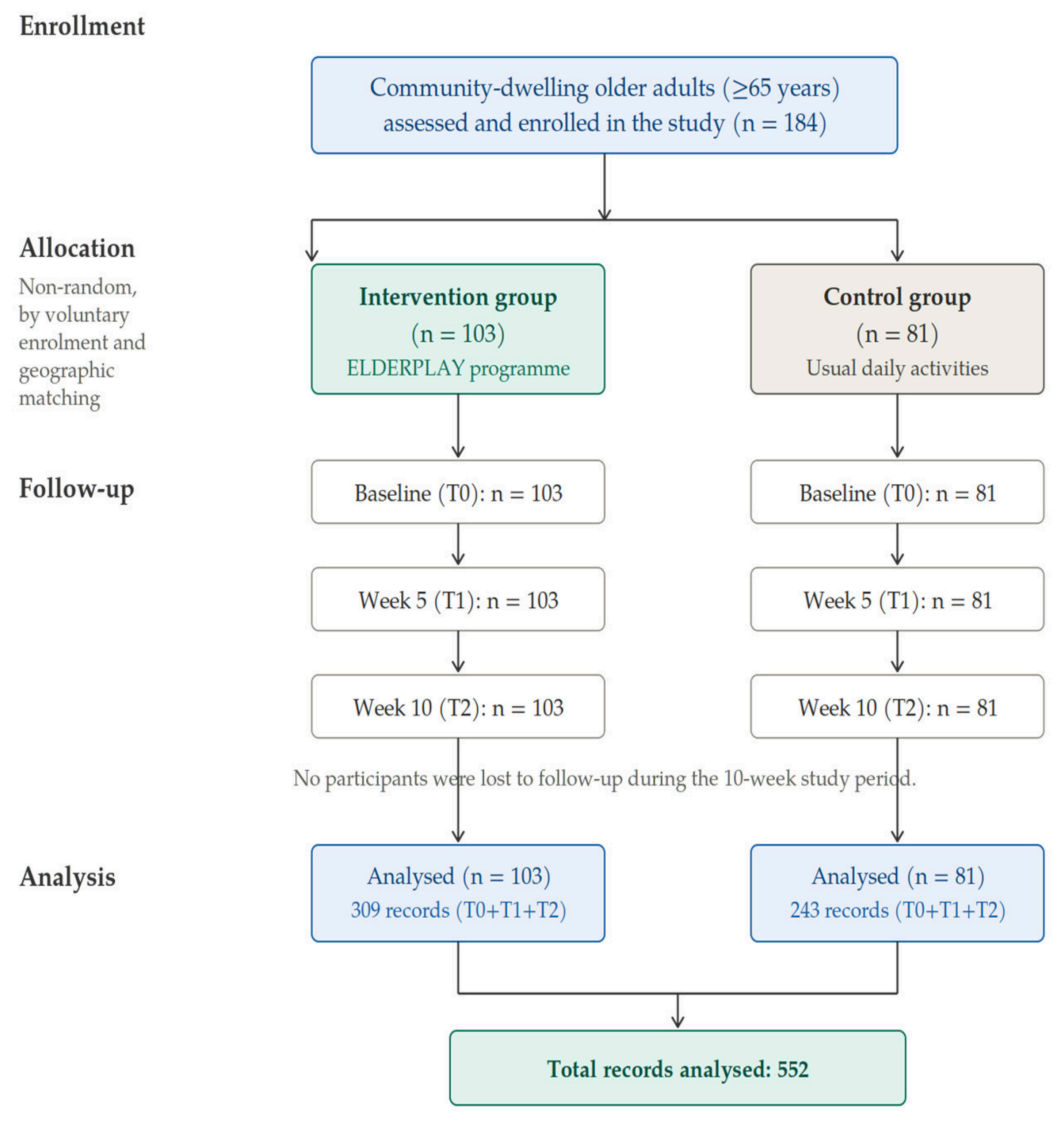
Flow of participants through the study (TREND diagram). Community-dwelling older adults aged 65 years or older were allocated to the intervention group (ELDERPLAY programme, n = 103) or the control group (usual daily activities, n = 81) through voluntary enrolment and geographic matching. Each participant was assessed at baseline (T0), at week 5 (T1) and at week 10 (T2). No participants were lost to follow-up, yielding 552 records (309 in the intervention group and 243 in the control group).

A total of 184 adults aged 65 or older residing in Lorca participated, with 81 in the control group and 103 in the intervention group. Each participant was evaluated at three points in time (start (T0), 5 weeks (T1), and at the end of the 10-week programme (T2)), resulting in 552 records (Figure 1). The sociodemographic and clinical characteristics are shown in Table 1. Most participants were women (88–91%), with an average age of 75 years, of Spanish nationality, and about 75% were overweight or obese. Notable differences appeared in marital status, with 62% married in the control group compared to 39% in the intervention group, and in education level, with 69% having no formal education in the control group versus 30% in the intervention group. The intervention group also showed a statistically significantly lower prevalence of diabetes (*p* = 0.044) and of high cholesterol (*p* = 0.046), together with a lower prevalence of chronic back pain that reached the conventional threshold only marginally (*p* = 0.050). Lastly, 13% of the intervention group had received the COVID-19 vaccine compared to 56% in the control group.

According to the Pfeiffer Test results, at the start of the study, 82.2% of the participants in the CG had a normal cognitive function, while 14.8% showed a mild cognitive deterioration; in the IG, these percentages were 86.4% and 13.6%, respectively, without statistically significant differences between the groups (*p* = 0.813). In contrast, significant differences were observed in the Barthel Index (*p* < 0.001): 56.8% of the CG and 88.3% of the IG demonstrated an independent level of functionality, while 42% of the CG and 11.7% of the IG presented mild dependency.

### 3.2. Balance Outcomes (SPPB)

**Figure 2:**
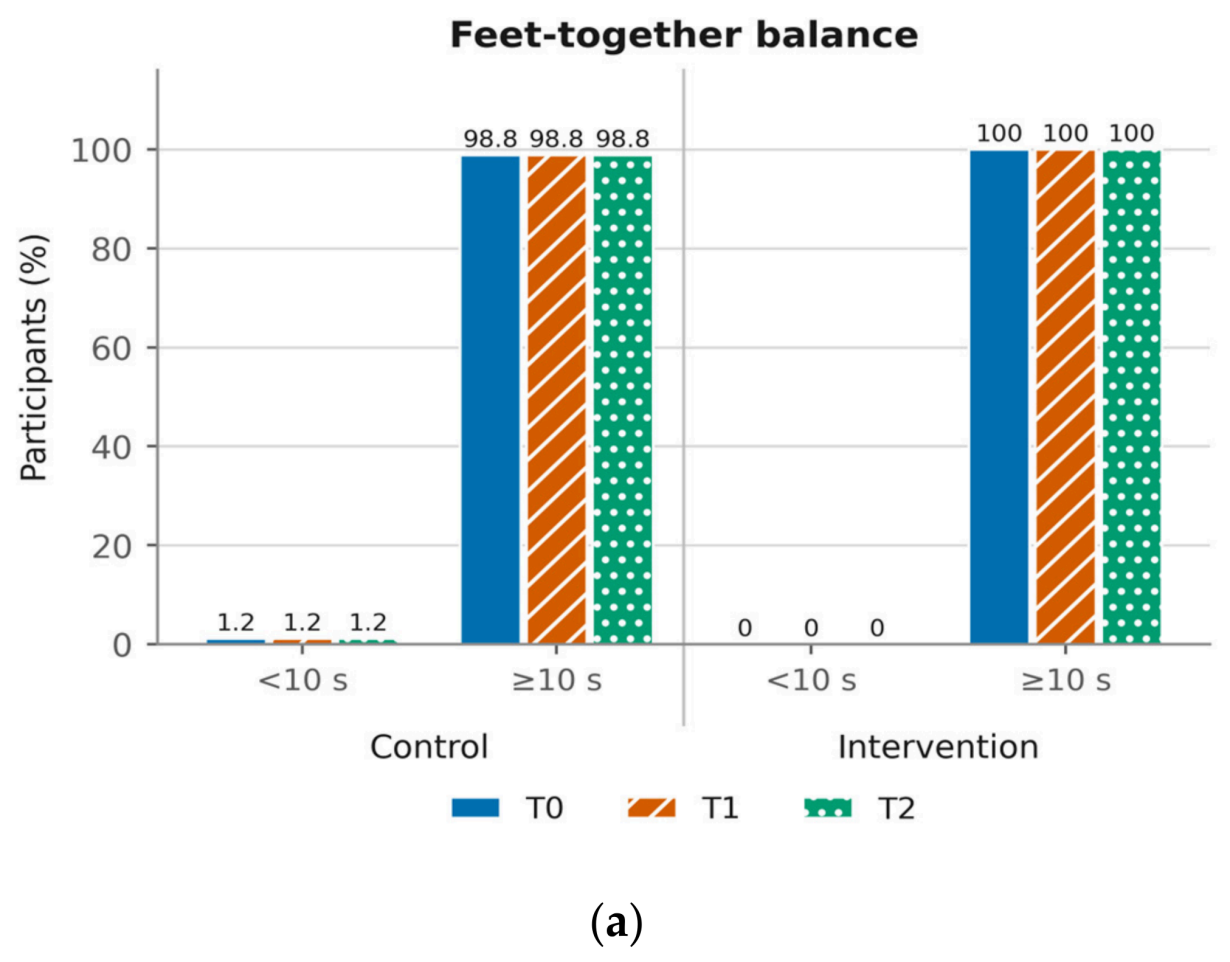

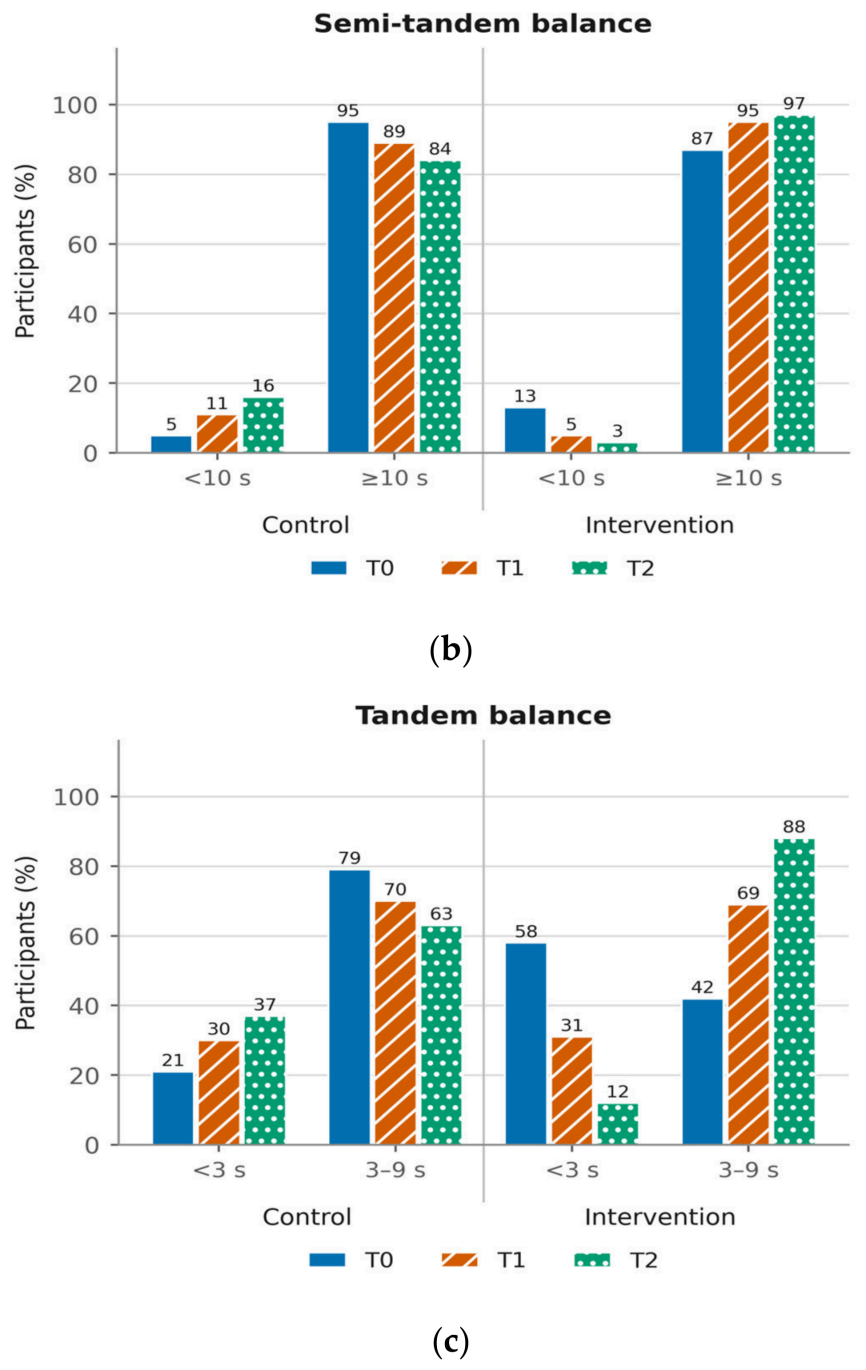
Short Physical Performance Battery (SPPB) balance test results: comparison of control and intervention groups across time (T0, T1, T2). (**a**) Feet-together balance test; (**b**) semi-tandem balance test; (**c**) tandem balance test. Bars represent the percentage of participants in each performance category at the three assessment points (T0, T1, T2), shown separately for the control and the intervention group.

With respect to the results directly associated with the impact of the intervention on frailty-related function, significant and positive effects in balance were observed in favour of the group that took part in the ELDERPLAY programme, assessed with the Short Physical Performance Battery (SPPB) (Figure 2). In all balance models the modelled event was achievement of the balance category specified in each case, and the control group was set as the reference category, so that odds ratios are expressed as intervention versus control. In the semi-tandem balance test, the model demonstrated a good fit (QIC = 316.969) and a significant interaction between group and moment (Wald χ^2^ = 16.395; *p* < 0.001), indicating differential change between groups: while the intervention group showed progressive improvement, the control group deteriorated. The estimations indicated that during the final measurement (T2), participants in the intervention group had significantly higher probabilities of maintaining balance (>10 s) compared to the control group (OR = 6.37; 95% CI: 1.75–23.26; *p* = 0.005). At the intragroup level, the control group showed a significant reduction in performance at every time point compared with baseline. In contrast, the intervention group showed improvements of 8% and 10% after 5 and 10 weeks, respectively.

Regarding the tandem balance test, a good fit to the model was also observed (QIC = 630.829), along with a significant interaction between the group and moment (Wald χ^2^ = 64.698; *p* < 0.001). At the end of the intervention (T2), subjects in the intervention group showed a significantly higher probability of maintaining balance (3–9 s) compared to the control group (OR = 4.46; 95% CI: 1.98–10.00; *p* < 0.001). The intragroup analyses revealed a progressive deterioration in the control group (up to −16% between T0 and T2), and a substantial improvement in the intervention group, with an increase of 27% in T1 and 46% in T2.

### 3.3. Gait Performance, Chair-Stand Performance, SPPB Total Score and Timed Up and Go

**Figure 3:**
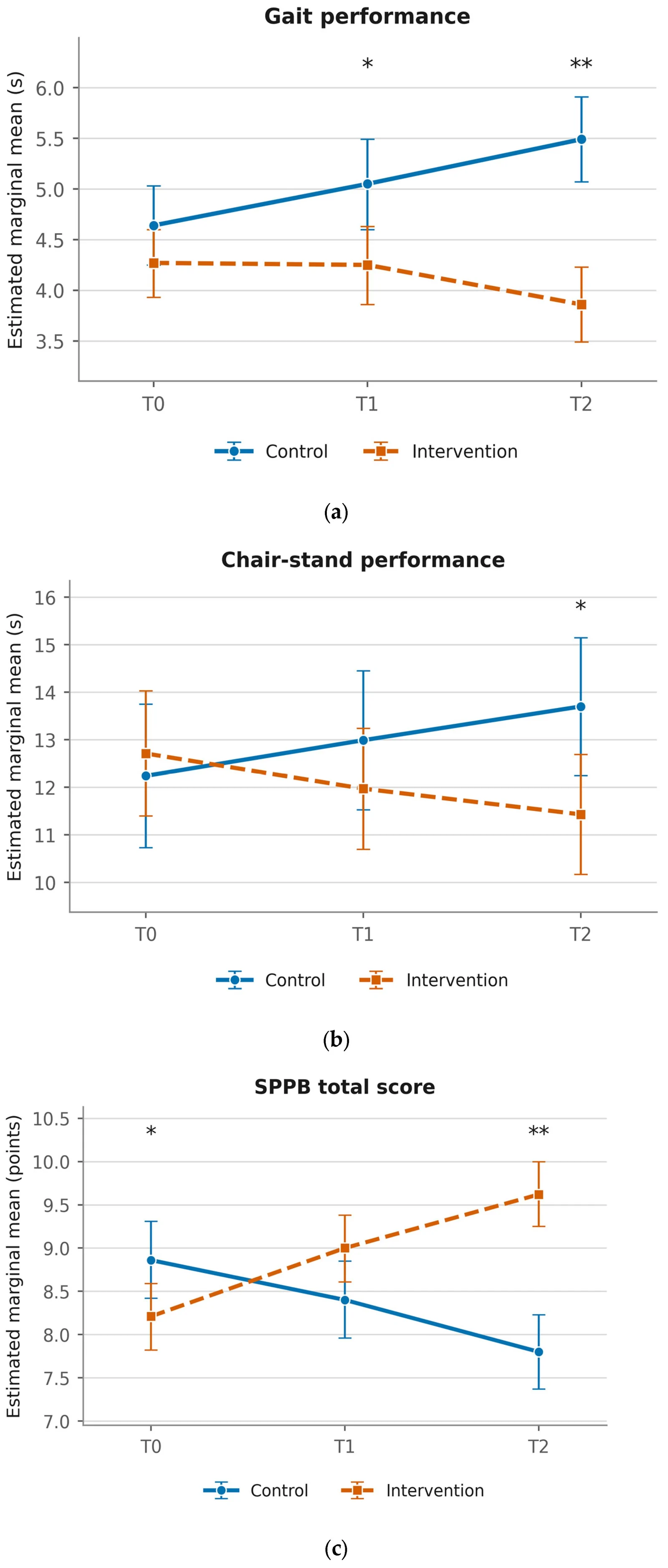

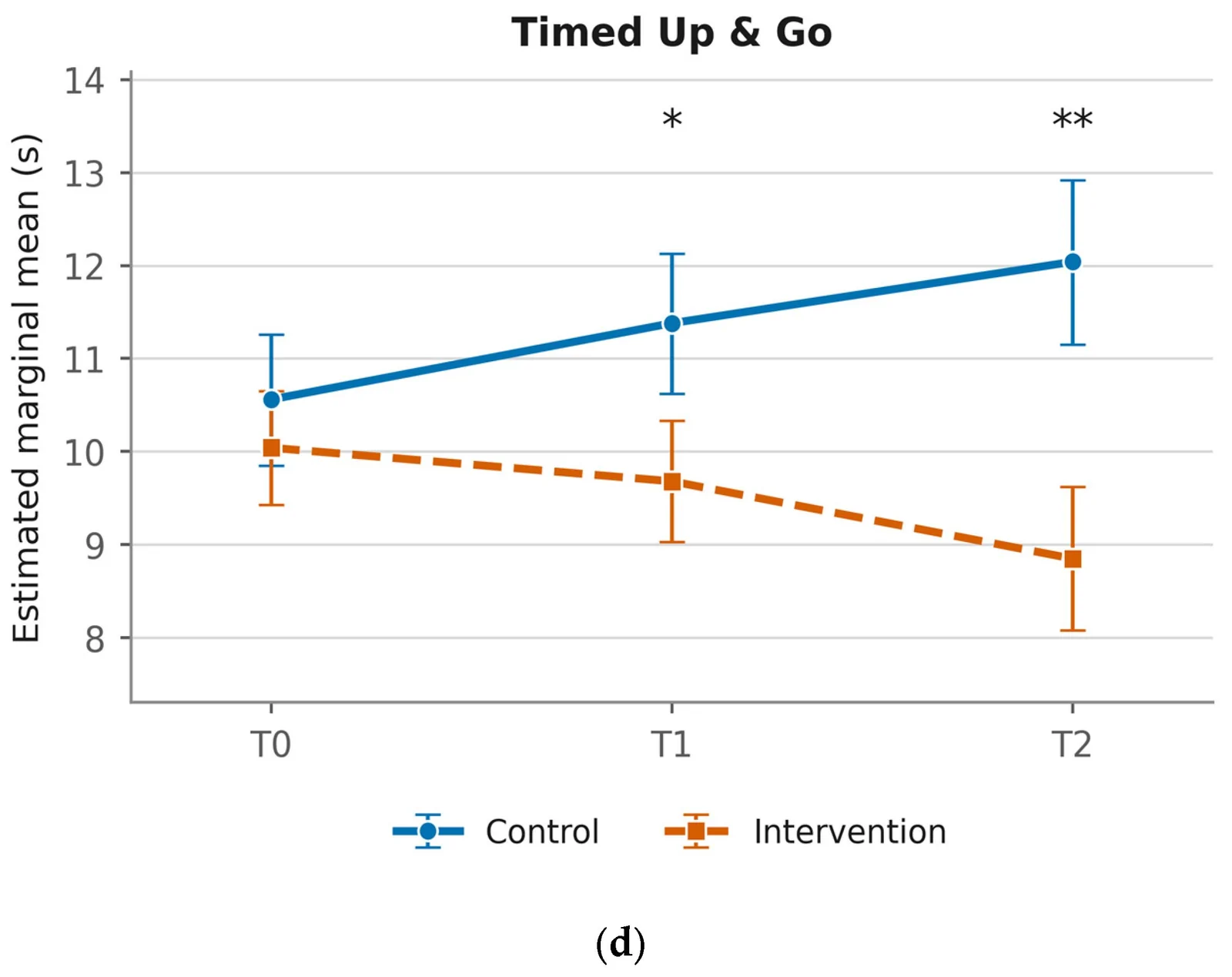
Longitudinal comparison of physical performance metrics (SPPB and Timed Up and Go) between control and intervention groups. (**a**) Gait performance (4 m walk time, s); (**b**) chair-stand test time (s); (**c**) SPPB total score; (**d**) Timed Up and Go (s). Values are estimated marginal means at T0, T1 and T2, adjusted for cholesterol, COVID-19 vaccination and the Barthel Index; error bars represent 95% confidence intervals. * *p* < 0.05 and ** *p* < 0.001 for the between-group comparison at that assessment point.

Table 2 and Figure 3 present the results of the comparison between the two groups with respect to gait performance, chair-stand performance, total SPPB score, and TUG. As for gait performance, a significant effect of time (*p* < 0.001; η^2^ = 0.101) and the group (*p* = 0.002; η^2^ = 0.053) was observed, as well as a highly significant interaction between time and intervention (F(2,178) = 72.900; *p* < 0.001; η^2^ = 0.450), which indicates a differential change between the groups. While the intervention group progressively reduced its 4 m walk time from 4.27 s at T0 to 3.86 s at T2, the control group showed a deterioration, increasing from 4.64 s to 5.49 s.

In the stand-up-from-a-chair test, the interaction between time and intervention was significant (F(2,178) = 39.327; *p* < 0.001; η^2^ = 0.306), indicating that the change over time differed between groups; the between-subjects effect of group did not reach statistical significance (*p* = 0.354). The intra-subject analysis revealed significant changes in both groups: the control group (*p* < 0.001; η^2^ = 0.226) and the intervention group (*p* < 0.001; η^2^ = 0.185) demonstrated varied performance over time. The CG increased from 12.24 to 13.70 s, while the IG decreased from 12.71 to 11.43 s. In the third measurement, significant differences were observed between groups (*p* = 0.032), favouring the intervention group.

The results from the total SPPB analysis showed a significant interaction between time and intervention (F(2,178) = 101.331; *p* < 0.001; η^2^ = 0.361), confirming differential changes between the groups. The IG improved from 8.21 to 9.62 points, while the CG showed a decrease from 8.86 to 7.80, with a difference close to statistical significance between groups (*p* = 0.054; η^2^ = 0.021). Both groups showed significant changes over time, with a higher impact in the intervention group (*p* < 0.001; η^2^ = 0.435) as compared to the control group (*p* < 0.001; η^2^ = 0.259). In the first assessment, the control group showed a better performance (*p* = 0.043), but this trend was reversed in the final measurement, in which the intervention group showed a significant improvement (*p* < 0.001).

The Timed Up and Go (TUG) test showed a significant interaction between time and intervention (F(2,178) = 25.656; *p* < 0.001; η^2^ = 0.224). The analysis between subjects showed significant differences in favour of the intervention group (*p* = 0.001; η^2^ = 0.058), with a clearly divergent change: while the control group progressively worsened (changing from 10.56 to 12.04 s), the intervention group improved until a final mean of 8.85 s.

### 3.4. Sleep Quality and Actigraphy-Derived Parameters

As for sleep quality, although no statistically significant differences were observed in the Pittsburgh Sleep Quality Index (PSQI), the intervention group tended to show better results. Of these, 58.3% showed sleeping problems as compared to 64.4% in the control group (*p* = 0.321), and the PSQI mean was slightly higher in the control group, 7.84 (SD = 4.94), while in the intervention group, it was 6.59 (SD = 3.92) (*p* = 0.08).

Regarding the physiological parameters measured using actigraphy (Table 3), a statistically significant difference in heart rate was observed between the groups. The intervention group presented significantly higher values in maximum heart rate (149.04 ± 13.77 as compared to 110.9 ± 24.69; *p* < 0.001) and the mean (99.14 ± 6.76 as compared to 83.07 ± 12.95; *p* < 0.001), and a lower minimum heart rate (49.24 ± 2.75 as compared to 55.25 ± 7.85; *p* < 0.001). Likewise, significant differences were observed in the mean number of daily steps, with a higher level of physical activity in the intervention group (5768.73 ± 2442.55 steps) compared to the control group (4521.17 ± 1776.12 steps; *p* < 0.001). These recordings were obtained at a single time point (T1), in the middle of the programme, so that the seven-day recording week of the intervention group included two programme sessions. The higher maximum and mean heart rates, and part of the difference in daily step count, therefore reflect participation in the sessions during the recording week rather than a change in habitual physical activity or in cardiovascular or autonomic function. Two values additionally suggest measurement artefact: a mean maximum heart rate of 149.04 bpm in a sample with a mean age of 75 years is close to the age-predicted maximum, and the standard deviation of the minimum heart rate in the intervention group (2.75 bpm) is implausibly small for a between-subject distribution.

With respect to the total sleep time, both groups showed similar values, and the distribution of sleep did not show significant differences in the phases of light sleep (*p* = 0.124) and deep sleep (*p* = 0.493); however, significant differences were observed in REM sleep, with it being higher in the intervention group (6.27 ± 9.74 min) as compared to the control group (3.21 ± 7.22 min; *p* < 0.001), as well as the time spent napping (13.81 ± 23.47 min compared to 6.91 ± 16.68 min; *p* < 0.001). The REM sleep values obtained are physiologically implausible, since REM sleep normally accounts for approximately 20–25% of total sleep time, which in this sample would correspond to some 80–105 min per night. They therefore reflect misclassification by the device rather than a true difference between groups, and the same caution applies to the recorded nap time; both are reported for completeness and are not used to support any conclusion.

### 3.5. Programme Delivery Costs

The total cost of delivering the programme was €9503. Considering the 103 participants who actually took part, the mean cost was €92.3 per participant, which is reported here as the main result. The figure of €91.11 shown in the last column of Table 4 corresponds to a standard group of 15 attendees across the 20 sessions of the programme and is a unit reference for planning purposes, not the observed cost. The Xiaomi Mi Smart Band 6 devices (€3090, approximately one third of the total) were used exclusively for outcome measurement and are not required for routine delivery of the programme; they therefore constitute a research cost rather than a delivery cost. Excluding them, the cost of delivering the programme was €6413, equivalent to €62.3 per participant (Table 4).

## 4. Discussion

The study was conducted on a sample of 184 older adults residing in the municipality of Lorca, with a mean age of 75 years and a clear predominance of women, possibly related to greater social involvement in health-related activities [31]. Most of the participants were of Spanish nationality, with a low level of education, normal cognitive function, and a degree of functionality ranging from independent to mildly dependent. This was a self-selected convenience sample whose characteristics were comparable with those described in previous studies [32], but it cannot be assumed to be representative of the local older population.

The results showed that the ELDERPLAY programme was associated with better outcomes among the older adults who participated in the intervention, who showed significantly higher scores in the total SPPB, as well as in its three components—balance, gait performance, and chair-stand performance—aside from a better performance in the TUG test, as compared to the control group, which showed a decline in performance in these tests after 10 weeks of follow-up. The partial eta squared values associated with these differences (0.224–0.450) are large for a ten-week community programme and, in a self-selected sample with baseline differences in functional status, are likely to be inflated by selection and by regression to the mean; effect sizes of this magnitude should not be expected to be reproduced in a randomized trial. The magnitude of the change observed in the control group requires comment. Over only ten weeks, its SPPB total score fell by approximately 1.1 points, its 4 m gait time increased by 0.85 s and its Timed Up and Go time by 1.5 s. A decline of this size is not plausible as a natural trajectory in community-dwelling older adults in the absence of an intercurrent event, and should not be interpreted as one. Three non-exclusive explanations are more likely. First, measurement bias: outcome assessment was performed by a single assessor who was aware of group allocation and of the study hypothesis, and performance-based tests administered without blinding are susceptible to systematic differences in instruction, encouragement and timing. Second, regression to the mean, which in a self-selected sample recruited concurrently but not randomized may operate in opposite directions in the two groups. Third, baseline imbalance: the control group included a higher proportion of participants with mild dependency according to the Barthel Index and a markedly lower educational level, and both may have influenced initial performance and the trajectory observed. The between-group differences reported here should therefore be read as an upper bound rather than as an estimate of the effect that a randomized trial would be expected to find. Differences were also observed in the number of daily steps and in heart rate parameters; no significant difference was found in total sleep time. Lastly, the programme was delivered in full, comprising 20 sessions over 10 weeks, at a mean delivery cost of €92.3 per participant and with no losses at any of the three assessment points. No feasibility outcomes were prespecified, so these figures describe programme delivery rather than establishing feasibility in a formal sense.

To our knowledge, this is the first study to examine a physical and recreational activity programme that adapts traditional games, such as musical chairs, steal the bacon, and tic-tac-toe, as part of physical training on health and functionality indicators in older adults living in the community. The results showed an association with better physical function in older adults, beyond the gains in cognitive and social skills, inclusion and teamwork that have been previously studied [16]. ELDERPLAY is, however, a combined intervention: its design does not allow the contribution of the recreational component to be separated from that of the structured multicomponent exercise or from the social interaction inherent in a group format, so the improvements cannot be attributed to any single component.

Balance was significantly improved, with an upward increase observed in the proportion of subjects who were able to maintain the tandem position for 3–9 s, increasing by 27% at the 5-week mark and by 46% at week 10 of the intervention. Similar results were observed with other types of physical activity, such as resistance training, aerobic exercise, balance training, Tai Chi, yoga, and multicomponent programmes [33]. Likewise, a significant difference was observed in dynamic balance, as measured by the TUG test under standardized conditions [34]. The intervention group improved its time to 8.85 s, while the control group worsened to 12.04 s, coming close to the critical point established at 12.6 s, which indicates the existence of a high risk of falling [35]. The recreational activities, such as steal the bacon, musical chairs or petanque, involve movements such as balancing on one foot, standing up from a chair, jumping to specific coordinates, selecting an object and returning to the starting point, performed alongside other participants. These demands are consistent with the pattern of results observed, although the design does not allow the contribution of the recreational component to be distinguished from that of the structured multicomponent exercise performed within the same sessions. The differences in balance may relate not only to greater lower-limb capacity and agility, but also to cognitive factors, which have been demonstrated to be associated with positive balance results [36].

Although gait speed tends to be one of the indicators of physical functionality that shows the least response to physical training in older adults [37], significant improvements were observed in the intervention group in the present study. More specifically, this group reduced its 4 m walk time by 0.41 s, while the control group showed a deterioration, increasing its time by 0.85 s. These results acquire special relevance when considering that the participants from both groups showed factors that were previously associated with a lower gait speed, such as a low level of education, a high prevalence of overweight and obesity, hypercholesterolaemia, and chronic back pain [38].

In agreement with these results, the Short Physical Performance Battery (SPPB) showed a significant improvement in the intervention group, which reached a mean score of 9.62, as compared to 7.80 points in the control group. Given that a score below 10 on this scale is considered indicative of frailty [8], these results suggest that the intervention was associated not only with improved physical functionality, but also with bringing the participants closer to a clinically relevant threshold of functional recovery. In addition, in both cases, an increase or worsening of 1 point or more was observed, which corresponds to the minimum clinically important difference established for this instrument and is associated with the ability to perform tasks of daily life and the maintenance of independence [39].

The ELDERPLAY programme showed a reduction of 1.28 s in the standing-up-from-a-chair test, while the control group worsened by 1.46 s. Better performance on this test is commonly interpreted as reflecting greater lower-limb capacity [40]. The chair-stand test is nevertheless a functional performance measure influenced by strength, balance, coordination, movement strategy and confidence, and no direct measure of muscle strength was obtained in this study, so no inference regarding muscle strength can be drawn from these data. A relationship has also been shown between performance in this test and the increase in the number of daily steps [40]. In agreement with this evidence, our study showed that the intervention group obtained a mean of 1300 steps more per day than the control group, coming close to the recommended threshold of 6000 daily steps for older adults [41].

As for other health-related parameters, differences in heart rate were observed between groups. These differences should not be interpreted as evidence of cardiovascular or autonomic adaptation. The actigraphy week of the intervention group included two programme sessions, so the higher maximum and mean values largely capture the acute response to exercise performed during the recording period, and the same applies in part to the difference in daily step count. In addition, the mean maximum heart rate recorded is close to the age-predicted maximum for this age group, and the dispersion of the minimum heart rate is too small to be credible as a between-subject distribution; both observations point to limitations of the consumer-grade wrist-worn device rather than to physiological findings. No measure of heart rate variability was obtained and the comparison was cross-sectional, so autonomic modulation cannot be inferred from the available data. Aerobic and multimodal exercise programmes have been reported to improve autonomic control of the heart [42], but the present data can neither confirm nor refute such an effect. These parameters are therefore reported as descriptive values recorded during the programme and are not used to support any conclusion of this study.

We must interpret the sleep quality and sleep pattern results assessed with actigraphy with caution, as they were consecutively recorded over a period of 7 days, and causal relationships cannot be established. On the one hand, it was observed that both groups slept for seven hours on average, with mean PSQI scores higher than 5 points, and percentages between 58% and 65% of subjects with a low quality of sleep, figures similar to those reported in other studies conducted in older Spanish populations [43]. The intervention group showed higher values for REM sleep and napping time, but these comparisons were cross-sectional: actigraphy was recorded only at the intermediate assessment point, with no baseline or post-intervention measurement, so no change in sleep over the course of the programme was established. Previous work has reported associations between exercise programmes and sleep quality in older adults [44]; the present data can neither confirm nor refute such an association. In addition, sleep-stage classification derived from consumer-grade wrist-worn devices shows limited agreement with polysomnography, and no validation of the Mi Smart Band 6 for this purpose in older adults is available. These results are therefore exploratory and are not used to support any conclusion of this study.

Factors other than the physical training itself may account for part of the differences observed. Participants in the intervention group enrolled voluntarily in an existing community programme and may therefore have differed in motivation towards physical activity before baseline, a variable that was not measured. The programme was delivered in a group format within community organizations, so social engagement and group affiliation are plausible contributors in their own right. Finally, the groups differed at baseline in marital status, educational level, prevalence of chronic conditions and COVID-19 vaccination, and residual confounding cannot be excluded despite statistical adjustment. The differences observed should accordingly be read as associations rather than as effects attributable to the exercise component alone.

Lastly, the ELDERPLAY programme was delivered at a cost of between €62.3 and €92.3 per participant, depending on whether the research monitoring devices are included. These figures are delivery costs only. No cost-effectiveness or cost-utility analysis was performed, and no conclusion can therefore be drawn regarding economic return or potential savings for the health system. Falls were not measured in this study and no health-related quality-of-life utilities were collected, so neither an incremental cost-effectiveness ratio nor a cost-utility ratio can be estimated from these data; the prospective collection of falls and utility values is a design requirement of the randomized trial that this evaluation is intended to precede.

No participant was lost at any of the three assessment points over the ten weeks. This degree of retention is unusual for a community intervention of this size and duration and merits comment in its own right. Delivery in small groups within participants’ own neighbourhoods and rural districts, which reduced travel barriers, a stable instructor team throughout the programme, and the voluntary nature of participation, with no financial incentives, are plausible contributors. Two qualifications should nevertheless be noted: complete retention refers to the scheduled assessments and not to attendance at the twenty sessions, which was not recorded individually; and a fully retained, self-selected sample is likely to represent a particularly motivated population, which reinforces the limitation on generalizability.

### Study Limitations

The non-randomized design does not permit causal inference, and residual confounding, including unmeasured differences in prior physical activity or concurrent rehabilitation, cannot be excluded despite statistical adjustment; the findings should therefore be read as associations. The dose of the intervention actually received and the reproducibility of the training stimulus cannot be established with precision, and outcome assessment was not blinded to group allocation. Although all outcome assessments were performed by the same assessor in order to reduce inter-observer variability, assessor blinding was not feasible given the nature of the intervention, and the possibility of detection bias cannot be excluded. Covariates were selected on the basis of statistically significant between-group differences at baseline, a data-driven procedure that does not in itself guarantee adequate control of confounding and that is sensitive to the size of the sample. Educational level and marital status, which also differed markedly between the groups (Table 1), were not entered in the models: both are closely related to the functional and social profile of the participants, which is largely captured by the Barthel Index, and their inclusion would have increased the number of estimated parameters at the expense of model parsimony without a corresponding gain in explanatory capacity. Residual confounding by these variables therefore cannot be excluded.

The results do not support conclusions regarding muscle strength, cognitive function or falls, none of which were measured, nor regarding change over time in the device-derived variables, which are cross-sectional and, in the case of sleep, exploratory. The economic analysis reflects the cost of delivering the programme and not its cost-effectiveness. The study was not prospectively registered; registration was obtained retrospectively during the revision of this manuscript, which limits the protection that prospective registration affords against selective reporting of outcomes. Finally, the maintenance of the observed differences beyond ten weeks is unknown, and the characteristics of the sample limit the generalizability of the findings.

These considerations define the design of the randomized controlled trial that should follow this evaluation: random allocation, prospective measurement of baseline physical activity, systematic recording of attendance and compliance, blinded outcome assessment, objective cognitive assessment, prospective collection of falls and health-related quality-of-life data, multicentre recruitment, and follow-up beyond the end of the intervention.

## 5. Conclusions

Participation in the ELDERPLAY programme, which combines structured multicomponent exercise with adapted traditional games, was associated with better physical performance in community-dwelling older adults over 10 weeks. Compared with the control group, the intervention group showed greater improvement in balance, gait performance, chair-stand performance and overall Short Physical Performance Battery and Timed Up and Go scores, and a higher daily step count was also observed. The programme was delivered in full at a mean cost of €92.3 per participant from the perspective of the programme provider, or €62.3 per participant when the research monitoring devices are excluded. Whether this delivery cost would be sustainable within routine municipal and primary care services was not evaluated in this study. A randomized controlled trial with longer follow-up is required to confirm these results.

## Figures and Tables

**Table 1:** Sociodemographic and clinical characteristics of the participants by study group.

		Control Group	Intervention Group	*p*-Value
Age (M; SD)		74.77 (5.18)	74.89 (5.3)	0.700
Sex n (%)	Male	7 (8.6)	12 (11.7)	0.506
	Female	74 (91.4)	91 (88.3)	
Nationality n (%)	Spanish	80 (98.8)	103 (100)	0.440
	Foreign	1 (1.2)	0 (0)	
Marital status n (%)	Single	4 (4.9)	14 (13.6)	0.008
	Married	50 (61.7)	40 (38.8)	
	Divorced or separated	1 (1.2)	5 (4.9)	
	Widowed	26 (32.1)	41 (39.8)	
	Widowed with a current partner	0 (0)	3 (2.9)	
Number of children (M; SD)		2.26 (1.18)	2.42 (1.33)	0.402
Number of grandchildren (M; SD)		3.15 (2.57)	3.50 (3.08)	0.416
Household composition n (%)	Live alone	30 (37)	42 (40.8)	0.038
	Live with a partner	45 (55.6)	43 (41.7)	
	Live with a partner and children	6 (7.4)	11 (10.7)	
	Live with children	0 (0)	7 (6.8)	
Level of education n (%)	No education	56 (69.1)	31 (30.1)	<0.001
	Primary education	17 (21)	63 (61.2)	
	Secondary education/vocational training	6 (7.4)	6 (5.8)	
	University degree	2 (2.5)	3 (2.9)	
Body Mass Index (M; SD)		27.62 (5.38)	27.47 (3.48)	0.348
Obesity n (%)	Low weight	2 (2.5)	0 (0)	0.132
	Normal weight	13 (16)	30 (29.1)	
	Overweight	42 (51.9)	50 (48.5)	
	Mild obesity 1	21 (25.9)	20 (19.4)	
	Average obesity 2	3 (3.7)	3 (2.9)	
	Morbid obesity 3	0 (0)	0 (0)	
Diabetes n (%)	Yes	29 (35.8)	23 (22.3)	0.044
High blood pressure n (%)	Yes	52 (64.2)	56 (54.4)	0.179
Heart disease n (%)	Yes	28 (34.6)	23 (22.3)	0.066
Cholesterol n (%)	Yes	45 (55.6)	42 (40.8)	0.046
Tobacco n (%)	Yes	1 (1.2)	5 (4.9)	0.231
Alcohol n (%)	Yes	18 (22.2)	16 (15.5)	0.246
Chronic pain (shoulder) n (%)	Yes	36 (44.4)	32 (31.1)	0.062
Chronic pain (back) n (%)	Yes	44 (54.3)	41 (39.8)	0.050
Chronic pain (knee) n (%)	Yes	41 (50.6)	43 (41.7)	0.231
Presence of chronic wounds n (%)	Yes	2 (2.5)	4 (3.9)	0.696
History of COVID-19 n (%)	Yes	75 (92.6)	95 (92.2)	0.927
COVID-19 vaccine n (%)	Yes	45 (55.6)	13 (12.6)	<0.001

M: mean; SD: standard deviation; N: number of participants; BMI: Body Mass Index. Categorical variables are reported as n (%). Weight categories were defined according to the World Health Organization classification of body mass index: underweight < 18.5 kg/m^2^; normal weight 18.5–24.9 kg/m^2^; overweight 25.0–29.9 kg/m^2^; obesity class I 30.0–34.9 kg/m^2^; obesity class II 35.0–39.9 kg/m^2^; obesity class III (morbid) ≥ 40.0 kg/m^2^.

**Table 2:** Comparison of mean scores on the Timed Up and Go and SPPB scales between control and intervention groups at T0, T1 and T2, and between subjects at T0, T1 and T2, adjusted for covariates.

		T0	T1	T2	Within-Subjects Effect of Time (B)			Between-Subjects Effect of Group (A)		
		M (95% CI)	M (95% CI)	M (95% CI)	*F*	*p*	*Partial η* ^2^	*F*	*p*	*Partial η* ^2^
**Gait performance (4 m walk time, s)**	CG ^b,c^	4.64 (4.25–5.03)	5.05 * (4.60–5.49)	5.49 ** (5.07–5.91)	58.008	<0.001	0.395	9.942	0.002	0.053
	IG	4.27 (3.93–4.60)	4.25 (3.86–4.63)	3.86 (3.49–4.23)	30.893	<0.001	0.258			
**Chair-stand test time (s)**	CG ^b,c^	12.24 (10.73–13.75)	12.99 (11.53–14.45)	13.70 * (12.25–15.15)	25.952	<0.001	0.226	0.864	0.354	0.005
	IG ^b,c^	12.71 (11.40–14.03)	11.97 (10.70–13.24)	11.43 (10.17–12.69)	20.218	<0.001	0.185			
**SPPB total score**	CG ^a,b,c^	8.86 * (8.42–9.31)	8.40 (7.96–8.85)	7.80 ** (7.37–8.23)	31.061	<0.001	0.259	3.753	0.054	0.021
	IG ^a,b,c^	8.21 (7.82–8.59)	9.00 (8.61–9.38)	9.62 (9.25–10.00)	68.505	<0.001	0.435			
**Timed Up and Go (s)**	CG ^a,b,c^	10.56 (9.85–11.26)	11.38 * (10.62–12.13)	12.04 ** (11.15–12.92)	18.923	<0.001	0.175	10.943	0.001	0.058
	IG ^a,b,c^	10.04 (9.43–10.65)	9.68 (9.03–10.33)	8.85 (8.08–9.62)	14.534	<0.001	0.140			

s: seconds; M: mean; 95% CI: 95% confidence interval; η^2^: eta squared; IG: intervention group; CG: control group; T0: time 0; T1: time 1; T2: time 2; SPPB: Short Physical Performance Battery. * *p* < 0.05 comparison IG vs. CG; ** *p* < 0.001 comparison IG vs. CG. ^a^ *p* < 0.05 comparison T0 vs. T1; ^b^ *p* < 0.001 comparison T1 vs. T2; ^c^ *p* < 0.001 comparison T0 vs. T2. Covariates: cholesterol, COVID-19 vaccination, Barthel Index.

**Table 3:** Actigraphy results by study group.

		Control Group	Intervention Group	*p*-Value
Heart rate (bpm) M (SD)	Maximum HR	110.9 (24.69)	149.04 (13.77)	<0.001
	Minimum HR	55.25 (7.85)	49.24 (2.75)	<0.001
	Mean HR	83.07 (12.95)	99.14 (6.76)	<0.001
Daily steps M (SD)		4521.17 (1776.12)	5768.73 (2442.55)	<0.001
Sleep time (min) M (SD)		404.98 (61.44)	416.21 (83.59)	0.39
Distribution of sleep (min) M (SD)	Light sleep	322.81 (45.23)	330.8 (71.32)	0.124
	Deep sleep	78.95 (32.16)	79.13 (32.35)	0.493
	REM sleep	3.21 (7.22)	6.27 (9.74)	<0.001
	Naps	6.91 (16.68)	13.81 (23.47)	<0.001

M: mean; SD: standard deviation; HR: heart rate; bpm: beats per minute; REM: rapid eye movement.

**Table 4:** Estimated cost per participant of the ELDERPLAY programme.

Concept	Session Price (1 h; Max. 15 per Group)	Total Session Price (20 h)	Participant Price	Total Intervention Price (103 Users; 140 Sessions)
Central court rental (San José Pavilion)	€20	€400	€26.7	€2800
Gerontogymnastics instructor (60 h per month); gross salary €615; gross + contributions €865	€14.41	€288	€19.21	€2017.4
Travel: €0.21 per kilometre; average 40 km	€8.4	€168	€11.2	€1176
Actigraphy bracelet (Xiaomi Mi Smart Band 6)	—	—	€30	€3090
Consumables	€3	€60	€4	€420
		**TOTAL**	**€91.11**	**€9503**

€: euros; h: hours. Costs were estimated using an activity-based costing approach. The participant price column expresses the cost per participant of a standard group of 15 attendees across the 20 sessions of the programme, whereas the total intervention price column refers to the programme as actually delivered (103 participants distributed across seven parallel groups, 140 sessions in total); the two columns therefore use different denominators and one is not the direct product of the other.

## Data Availability

The data presented in this study are available on request from the corresponding author due to privacy restrictions concerning participant confidentiality.

## Supplementary Materials

The following supporting information can be downloaded at: https://www.mdpi.com/article/10.3390/healthcare14203441/s1, Table S1: TREND Statement checklist; Table S2: Description of the adapted traditional games used in the ELDERPLAY programme. Reference [45] is included in the Supplementary Materials.

## Abbreviations

The following abbreviations are used in this manuscript:

ABC	Activity-Based Costing
ANCOVA	Analysis of covariance
BMI	Body Mass Index
CG	Control group
CI	Confidence interval
GEE	Generalized Estimating Equations
HR	Heart rate
IG	Intervention group
OR	Odds ratio
PSQI	Pittsburgh Sleep Quality Index
QIC	Quasi-likelihood under Independence Model Criterion
REM	Rapid eye movement
SD	Standard deviation
SPPB	Short Physical Performance Battery
TREND	Transparent Reporting of Evaluations with Nonrandomized Designs
TUG	Timed Up and Go
WHO	World Health Organization
