# Supplementary material for: Physical Performance Outcomes and Programme Delivery Costs of ELDERPLAY, a Community-Based Multicomponent Exercise and Recreational Intervention for Older Adults: A Non-Randomized Controlled Evaluation
Source: Healthcare (Basel). 2026 Oct 14;14(20):3441. doi: 10.3390/healthcare14203441 (PMC13704763; doi:10.3390/healthcare14203441)
Supplement: Supplementary file 1 [file healthcare-14-03441-s001.zip › healthcare-4490605-supplementary.pdf]

Table S1: TREND Statement Checklist
Transparent Reporting of Evaluations with Nonrandomized Designs

Manuscript: Physical Performance Outcomes and Programme Delivery Costs of ELDERPLAY, a Community-Based Multicomponent Exercise and Recreational Intervention for Older Adults: A Non-Randomized Controlled Evaluation

Reporting statement: This manuscript adheres to the TREND Statement.

Paper Section/Topic	Item No	Descriptor	Reported?	
			✓	Pg #
**Title and Abstract**				
Title and Abstract	1	• Information on how unit were allocated to interventions	Yes	1–2
		• Structured abstract recommended	Yes	1–2
		• Information on target population or study sample	Yes	1–2
**Introduction**				
Background	2	• Scientific background and explanation of rationale	Yes	3–5
		• Theories used in designing behavioral interventions	N/A	
**Methods**				
Participants	3	• Eligibility criteria for participants, including criteria at different levels in recruitment/sampling plan (e.g., cities, clinics, subjects)	Yes	6
		• Method of recruitment (e.g., referral, self-selection), including the sampling method if a systematic sampling plan was implemented	Yes	6
		• Recruitment setting	Yes	6
		• Settings and locations where the data were collected	Yes	5–6
Interventions	4	• Details of the interventions intended for each study condition and how and when they were actually administered, specifically including:		7–8
		○ Content: what was given?	Yes	7–8
		○ Delivery method: how was the content given?	Yes	7–8
		○ Unit of delivery: how were the subjects grouped during delivery?	Yes	7–8

		○ Deliverer: who delivered the intervention?	Yes	7–8
		○ Setting: where was the intervention delivered?	Yes	7–8
		○ Exposure quantity and duration: how many sessions or episodes or events were intended to be delivered? How long were they intended to last?	Yes	7–8
		○ Time span: how long was it intended to take to deliver the intervention to each unit?	Yes	7–8
		○ Activities to increase compliance or adherence (e.g., incentives)	Yes	7–8
Objectives	5	• Specific objectives and hypotheses	Yes	5
Outcomes	6	• Clearly defined primary and secondary outcome measures	Yes	8–10
		• Methods used to collect data and any methods used to enhance the quality of measurements	Yes	9–10
		• Information on validated instruments such as psychometric and biometric properties	Yes	8–9
Sample Size	7	• How sample size was determined and, when applicable, explanation of any interim analyses and stopping rules	Yes	6
Assignment Method	8	• Unit of assignment (the unit being assigned to study condition, e.g., individual, group, community)	Yes	5–6
		• Method used to assign units to study conditions, including details of any restriction (e.g., blocking, stratification, minimization)	Yes	6
		• Inclusion of aspects employed to help minimize potential bias induced due to non-randomization (e.g., matching)	Yes	6
Blinding (masking)	9	• Whether or not participants, those administering the interventions, and those assessing the outcomes were blinded to study condition assignment; if so, statement regarding how the blinding was accomplished and how it was assessed.	Yes (no blinding). The intervention was delivered by three trained sports instructors; outcome assessment was performed by a single unblinded	2, 9–10, 30

			assessor, the principal investigator, who did not deliver the sessions.	
Unit of Analysis	10	•Description of the smallest unit that is being analyzed to assess intervention effects (e.g., individual, group, or community)	Yes	11–12
		• If the unit of analysis differs from the unit of assignment, the analytical method used to account for this (e.g., adjusting the standard error estimates by the design effect or using multilevel analysis)	Yes	11–12
Statistical Methods	11	• Statistical methods used to compare study groups for primary outcome(s), including complex methods of correlated data	Yes	11–12
		• Statistical methods used for additional analyses, such as subgroup analyses and adjusted analysis	Yes	11–12
		• Methods for imputing missing data, if used	Yes	9, 12
		• Statistical software or programs used	Yes	12
**Results**				
Participant flow	12	• Flow of participants through each stage of the study: enrollment, assignment, allocation, and intervention exposure, follow-up, analysis (a diagram is strongly recommended)	Yes	14
		○ Enrollment: the numbers of participants screened for eligibility, found to be eligible or not eligible, declined to be enrolled, and enrolled in the study	Yes	14
		○ Assignment: the numbers of participants assigned to a study condition	Yes	14
		○ Allocation and intervention exposure: the number of participants assigned to each study condition and the number of participants who received each intervention	Yes	14
		○ Follow-up: the number of participants who completed the follow-up or did not complete the follow-up (i.e., lost to follow-up), by study condition	Yes	14, 30
		○ Analysis: the number of participants included in or excluded from the main analysis, by study condition	Yes	13–14

		• Description of protocol deviations from study as planned, along with reasons	Yes	30
Recruitment	13	Dates defining the periods of recruitment and follow-up	Yes	6
Baseline Data	14	• Baseline demographic and clinical characteristics of participants in each study condition	Yes	13, 15–16
		• Baseline characteristics for each study condition relevant to specific disease prevention research	Yes	15–16
		• Baseline comparisons of those lost to follow-up and those retained, overall and by study condition	Yes	14, 28
		• Comparison between study population at baseline and target population of interest	Yes	6, 13
Baseline equivalence	15	• Data on study group equivalence at baseline and statistical methods used to control for baseline differences	Yes	11–12, 15–16
Numbers analyzed	16	• Number of participants (denominator) included in each analysis for each study condition, particularly when the denominators change for different outcomes; statement of the results in absolute numbers when feasible	Yes	13–23
		• Indication of whether the analysis strategy was "intention to treat" or, if not, description of how non-compliers were treated in the analyses	Yes	11–12
Outcomes and estimation	17	• For each primary and secondary outcome, a summary of results for each study condition, and the estimated effect size and a confidence interval to indicate the precision	Yes	16–23
		• Inclusion of null and negative findings		23
		• Inclusion of results from testing pre-specified causal pathways through which the intervention was intended to operate, if any		
Ancillary analyses	18	• Summary of other analyses performed, including subgroup or restricted analyses, indicating which are pre-specified or exploratory	Yes (stratified sensitivity analyses by Barthel Index category; no pre-specified subgroup	12

			analyses)	
Adverse events	19	• Summary of all important adverse events or unintended effects in each study condition (including summary measures, effect size estimates, and confidence intervals)	Yes (none recorded)	13
**Discussion**				
Interpretation	20	• Interpretation of the results, taking into account study hypotheses, sources of potential bias, imprecision of measures, multiplicative analyses, and other limitations or weaknesses of the study	Yes	25–30
		• Discussion of results taking into account the mechanism by which the intervention was intended to work (causal pathways) or alternative mechanisms or explanations	Yes	25–30
		• Discussion of the success of and barriers to implementing the intervention, fidelity of implementation	Yes	25–30
		• Discussion of research, programmatic, or policy implications	Yes	30–31
Generalizability	21	• Generalizability (external validity) of the trial findings, taking into account the study population, the characteristics of the intervention, length of follow-up, incentives, compliance rates, specific sites/settings involved in the study, and other contextual issues	Yes	30
Overall Evidence	22	• General interpretation of the results in the context of current evidence and current theory	Yes	25–31

Source: *Des Jarlais, D. C., Lyles, C., Crepaz, N., & the TREND Group (2004). Improving the reporting quality of nonrandomized evaluations of behavioral and public health interventions: The TREND statement. American Journal of Public Health, 94, 361–366. For more information, visit: http://www.cdc.gov/trendstatement/*

Table S2. Description of the adapted traditional games used in the ELDERPLAY programme.

Traditional game	Description of the version used in the programme	Materials	Capacities primarily involved
Steal the bacon	Two teams stand facing each other in two lines. An object is placed in the centre and, when a number is called, the two participants with that number step forward to take it and return to their line without being tagged.	Foam baton or soft object; cones	Gait speed, reaction time, dynamic balance, selective attention
Musical chairs	Participants walk around a row of chairs while music plays and sit down when the music stops. In the adapted version the number of chairs is not reduced below the number of participants, so that no one is eliminated.	Chairs; music player	Sit-to-stand transfers, lower-limb functional performance, gait, auditory reaction
Relay races	Teams complete a walking course in turn,	Baton or ring; cones	Gait speed, dynamic balance, aerobic

Traditional game	Description of the version used in the programme	Materials	Capacities primarily involved
	handing over an object at the end of each leg. Distances are short and the course is free of obstacles.		endurance, cooperation
Tic-tac-toe	Two teams take turns walking to a floor grid and placing a marker in one of the nine squares, aiming to complete a line of three.	Floor grid (mat or tape); coloured markers	Gait, planning and spatial reasoning, upper-limb reach, trunk control
Petanque	Participants throw balls towards a small target ball, aiming to place them as close to it as possible.	Petanque or foam balls; target ball	Upper-limb coordination, aiming precision, trunk control, standing balance
Hopscotch	Participants advance along a numbered floor pattern. In the adapted version, jumping is replaced by stepping from square to square with support available if required.	Floor pattern (mat or tape); marker stone	Static and dynamic balance, weight transfer, sequencing, numerical processing
Red light, green light	Participants walk towards a goal and must stop immediately when the leader gives the signal, remaining still until walking is allowed again.	None	Gait initiation and termination, inhibitory control, static balance
Hide and seek	One participant looks for objects or partners hidden within a delimited area of the room, following verbal clues.	Cones or screens; small objects	Gait, visual search, orientation, spatial memory
Bowling	Participants roll a ball along the floor to knock down a set of pins placed at a short distance.	Plastic pins; soft ball	Upper-limb coordination, aiming precision, trunk rotation, standing balance

All games were adapted to the functional level of the participants and supervised by the instructors: running, physical contact and floor-level actions were replaced by walking versions or by seated or standing alternatives, and no participant was eliminated from a game. Groups were limited to 15 participants. [PENDIENTE: confirmar con el equipo las adaptaciones concretas aplicadas a cada juego y añadir cualquier actividad recreativa no recogida en esta tabla.]
